# Enhancing the functional output of transplanted islets in diabetic mice using a drug-eluting scaffold

**DOI:** 10.1186/s13036-018-0098-3

**Published:** 2018-04-18

**Authors:** Kelei Zhu, Leqi Dong, Jinbo Wang, Dingyao Li, Mingliang Chen, Cunbin Jiang, Jinfa Wang

**Affiliations:** 1 Department of Heptobiliary Surgery, Yinzhou People’s Hospital, Ningbo, Zhejiang China; 2 Department of Gastroenterology, Yinzhou People’s Hospital, Ningbo, Zhejiang China; 3 Yin Zhou Hospital, Medical School of Ningbo University, Baizhang Road 251, Ningbo, 315000 Zhejiang China

**Keywords:** Electrospun scaffold, Islet transplantation, Macrophages, Interleukin 4, Diabetic

## Abstract

**Background:**

Islet transplantation is increasingly used in the diabetic patients to control the blood glucose level. However, the functional output of transplanted islets remains hampered due to the local inflammation, loss of islets, etc. To that end, in this study we explored to enhance the functional output of transplanted islets in diabetic mice by employing a drug-eluting scaffold with a payload of interleukin 4 (IL-4).

**Results:**

According to the in vitro studies, the scaffold showed no cytotoxicity, a rapid release of IL-4 within a week and the IL-4 retained its bioactivity. During the 4-week time window after the islet transplantation, in vivo studies showed that the levels of blood insulin and C-peptide 2 in diabetic mice in the drug-eluting scaffold group significantly increased since week 2, which effectively reduced the blood glucose level. In addition, these mice demonstrated a stronger capability to withstand a rapid glucose spike as evidenced by the tolerance of sudden oral glucose challenge test result. A further mechanistic study suggested that the enhanced functional output could be attributed to the M2 polarization of macrophages as evidenced by the increase of CD163^+^/CD68^+^ macrophages in the islet tissues. A M2 polarization of macrophages is widely believed to exert an anti-inflammatory influence on local tissues, which could accelerate the resolution of local inflammation following the islet transplantation.

**Conclusion:**

Our study shed a new light on the hyperglycemia management of diabetic patients following the islet transplantation.

## Background

Despite decades of scientific and clinical endeavors, diabetes remains one of the deadliest diseases that inflict millions of patients in the world [1]. Unfortunately, a prolonged hyperglycemia ultimately causes a cascade of other disorders, such as hypertension, nephrotic disorders, foot ulcers, etc. [2–4]. In type I diabetes, pancreatic ß-cells that are responsible for insulin production are functionally compromised due to auto-immunological disorders, giving rise to hyperglycemia [5]. In light of this, the traditional treatment for type I diabetes is to restore the blood insulin level by the periodical injection of insulin or the surgical transplantation of functional islets [6]. Previous clinical studies showed that the islet transplantation yielded a better prognosis on the long-term basis than the insulin injection [7]. However, the functional output of transplanted islets is frequently hampered due to various factors [8]. Previous clinical research has shed some light on the underlying challenges. For example, the post-surgery survival and functional output of transplanted islets are compromised by the local inflammation, the unfavorable milieu of the renal capsule where the islets were freely injected into, and so forth [9].

In the last two decades, biomaterial scientists have made significant progress on recouping lost functions of diseased tissues by employing various biomedical materials and cells [10]. In these efforts, a tissue-engineered scaffold is used to afford a favorable milieu following the implantation of cells to support the cascade of biological, including adhesion, migration, proliferation and so forth [11]. Furthermore, the tissue-engineered scaffold is frequently tooled to carry a payload of bioactive molecules so that the local release of these molecules will bring about a therapeutic effect [12].

In this study, we attempted to tissue engineer an electrospun scaffold with a payload of interleukin 4 (IL-4). The drug-eluting scaffold was expected to provide a favorable milieu to the transplanted islets in the renal capsule. Meanwhile, the IL-4 was supposed to be released locally due to the degradation of the scaffolding materials and to accelerate the resolution of local inflammation. It was hypothesized that the suppressed inflammation would ultimately help enhance the functional output of the transplanted islets.

## Results

### Biophysical characterizations of the scaffold

**Fig. 1:**
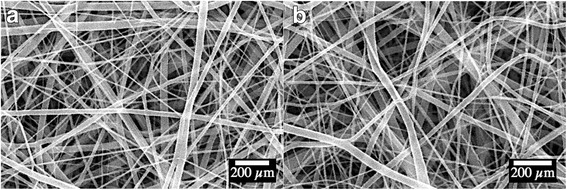
Morphological characterizations of electrospun scaffold. **a** drug-eluting scaffold; **b** non-eluting scaffold. No significant morphological difference was observed between drug-eluting and non-eluting scaffolds

The scanning electron microscope images showed that the electrospun scaffolds featured a highly porous microstructure composed of non-woven composite fibers (Fig. 1). The microstructure of drug-eluting scaffolds and non-eluting scaffolds showed no significant morphological difference, suggesting that the payload of IL-4 did not cause any structural changes. The absence of micro-structural changes in drug-eluting and non-eluting scaffolds eliminated the possibility that physical environmental cues, which had been shown to dictate cellular activities in tissue engineering, contributed to the different biological outputs of islet transplantations.

**Fig. 2:**
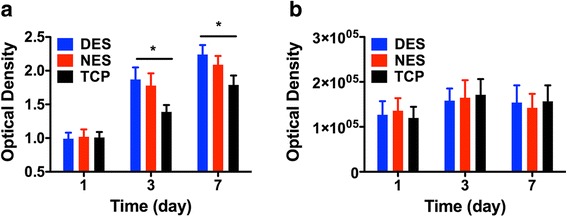
In vitro cytotoxic studies of electrospun scaffolds. **a** growth of islets cultured on electrospun scaffolds; **b** apoptosis of islets cultured on electrospun scaffolds. The scaffold supported the growth islets without triggering significant apoptosis. DES: drug-eluting scaffold (*n* = 4); NES: non-eluting scaffold (*n* = 4); TCP: tissue culture plate (control) (*n* = 4)

**Fig. 3:**
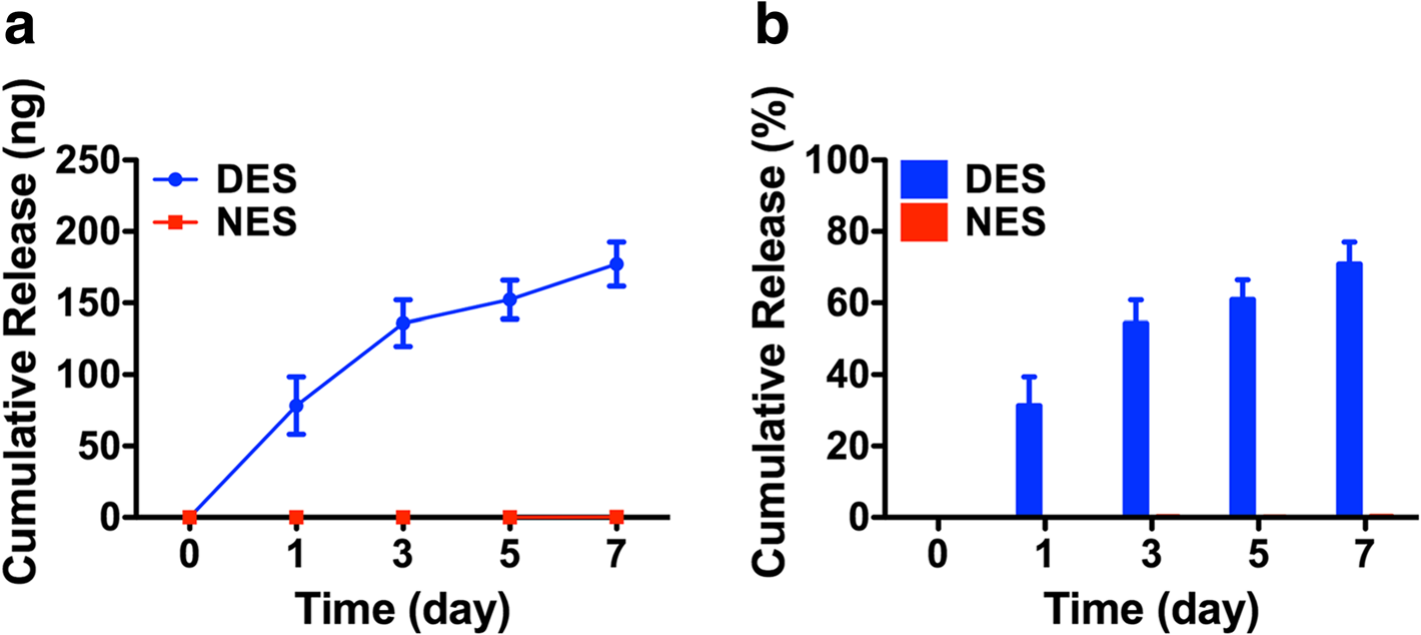
Pharmacokinetic study of IL-4 release. **a** the cumulative release amount; **b** the cumulative release percentage. The scaffold steadily released IL-4 in a 7-day time window. DES: drug-eluting scaffold (*n* = 4); NES: non-eluting scaffold (*n* = 4)

**Fig. 4:**
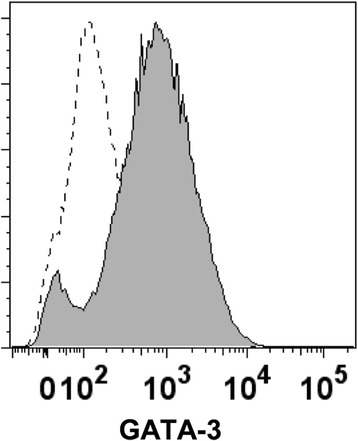
The bioactivity of released IL-4. The representative flow cytometric analysis confirmed that released IL-4 retained its capability of inducing the Th2 transformation of naïve CD4+ T cells as evidenced by the expression of GATA-3

According to the in vitro studies, the scaffolding materials showed no cytotoxicity as evidenced by a sustained proliferation of islets and a lack of significant apoptosis (Fig. 2). This lack of cytotoxicity indicated that these scaffolding materials were suitable for in vivo applications. Moreover, the pharmacokinetic study showed a sustained release of the IL-4 payload from the drug-eluting scaffold during a 7-day time window (Fig. 3). The cumulative release of IL-4 reached 177.21 ± 15.41 ng, accounting for 70.88% ± 6.16% of the loading amount. The in vitro bioactivity study showed that the released IL-4 successfully induced the transformation of naïve CD4^+^ T cells into Th2 phenotype as evidenced by the expression of GATA-3 (Fig. 4). This result confirmed that the released IL-4 retained its bioactivity for in vivo studies.

### The in vivo functional output of transplanted islets

**Fig. 5:**
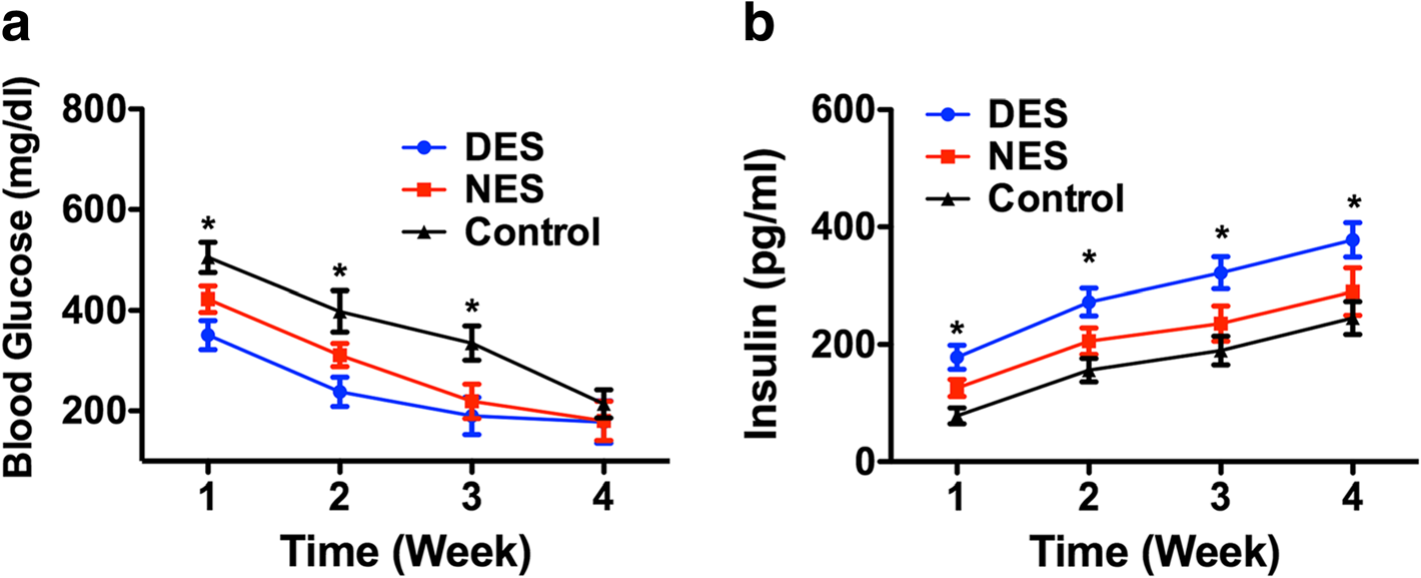
In vivo insulin production of transplanted islets. **a** blood glucose levels; **b** blood insulin levels. Mice from the drug-eluting scaffold enjoyed a higher blood insulin levels, which drove down the blood glucose level more rapidly. DES: drug-eluting scaffold (*n* = 5); NES: non-eluting scaffold (*n* = 4); Control: islets without scaffold (*n* = 6)

The blood glucose level in the drug-eluting scaffold group witnessed a significant decrease compared to that in the non-eluting scaffold group from week 2 after the islet transplantation (Fig. 5a). The blood glucose concentrations were 258 ± 27 mg/dL in the drug-eluting scaffold group and 339 ± 34 mg/dL in the non-eluting scaffold group, respectively. However, the difference of blood glucose levels between the two groups was not statistically significant at the end of week 4 after the islet transplantation. Serum insulin levels enjoyed a marked and sustained increase in the drug-eluting scaffold group ever since week 2 after the islet transplantation (Fig. 5b). The insulin concentrations were 291 ± 32 mg/dL in the drug-eluting scaffold group and 172 ± 18 mg/dL in the non-eluting scaffold group, respectively, at week 2 after the islet transplantation. These results suggested that the locally released IL-4 from the drug-eluting scaffold enhanced the insulin secretion capability of the transplanted islets, which successfully translated into a reduced blood glucose level.

**Fig. 6:**
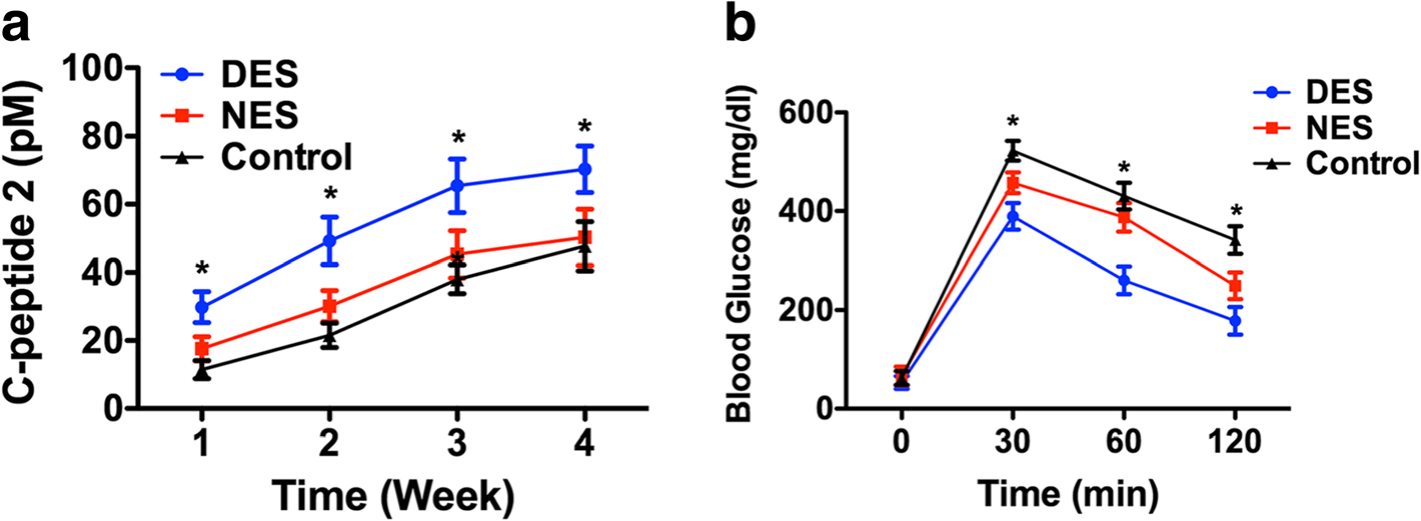
In vivo functional outputs of transplanted islets. **a** C-peptide 2; **b** OGTT. Mice from the drug-eluting scaffold showed a higher level of C-peptide and a stronger capacity to withstand sudden glucose challenge. DES: drug-eluting scaffold (*n* = 5); NES: non-eluting scaffold (*n* = 4); Control: islets without scaffold (*n* = 6)

**Fig. 7:**
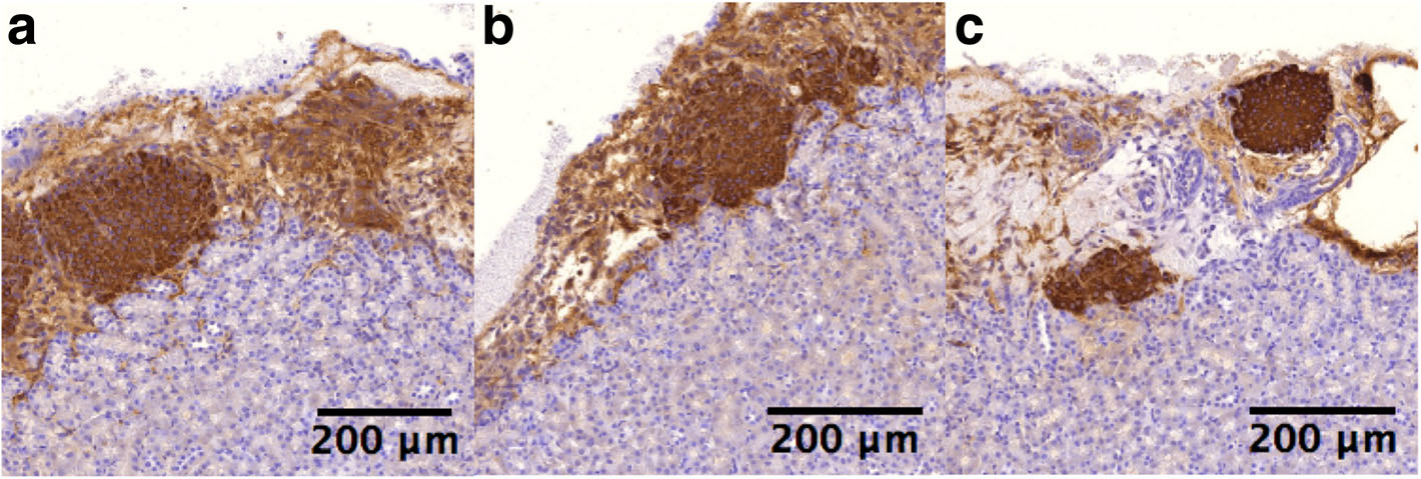
Immunohistochemistry staining of transplanted islets. **a** drug-eluting scaffold group; **b** non-eluting scaffold group; **c** control group

Furthermore, the C-peptide 2 levels in the drug-eluting scaffold group showed a significant increase starting from week 2 and enjoyed this heightened benefit through week 4 (Fig. 6a). The C-peptide 2 concentrations were 52.33 ± 9.27 pM in the drug-eluting scaffold group and 29.99 ± 4.98 pM in the non-eluting scaffold group, respectively, at week 2 after the transplantation. The oral glucose tolerance test (OGTT) test was conducted to gauge the capacity of diabetic mice to withstand glucose challenge following the islet transplantation. The result suggested that mice in the drug-eluting scaffold group exhibited a higher capacity of withstanding glucose challenge compared to their counterparts in the non-eluting scaffold groups (Fig. 6b). The blood glucose level in the mice from the drug-eluting scaffold group was 367 ± 27 mg/dL at 30 min after the oral gavage compared to 487 ± 49 mg/dL in the non-eluting scaffold. Throughout the 120-min time window, the blood glucose level in the mice from the drug-eluting scaffold group was consistently lower than those in the control group. The immunohistochemistry staining of retrieved islets in the renal capsule showed that insulin-secreting islets in the renal capsule (Fig. 7). This result confirmed that transplanted islets survived and was capable of secreting insulin.

### The polarization of macrophages in the transplantation site

**Fig. 8:**
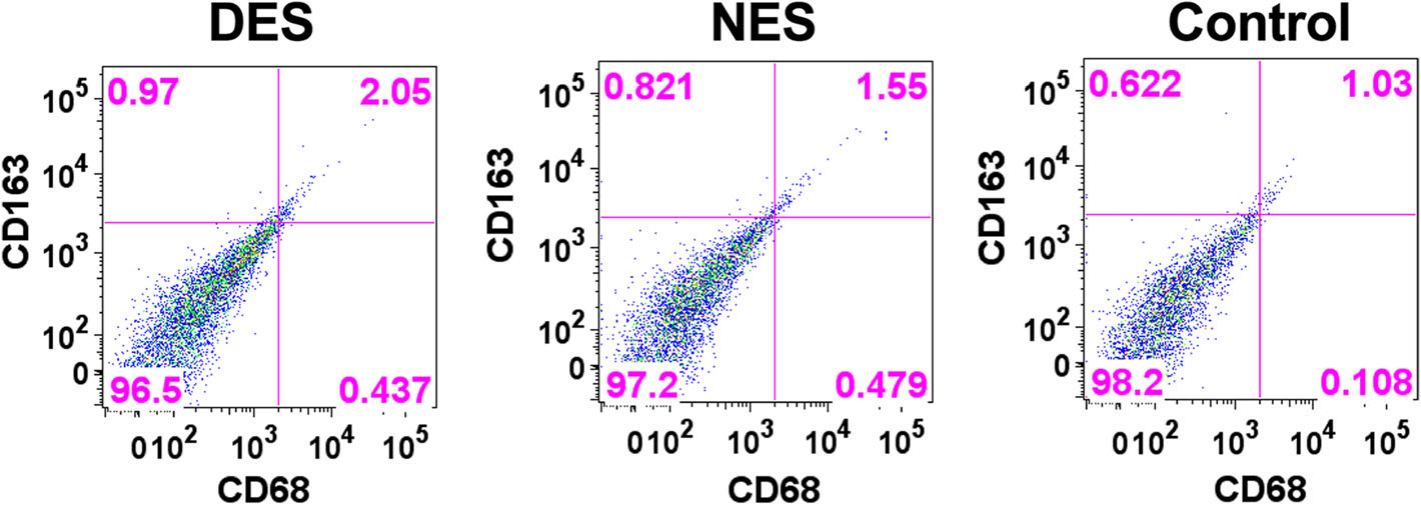
Representative flow cytometric chart of CD163^+^CD68^+^ M2 macrophages. Mice in the drug-eluting scaffold group showed a higher percentage of CD163^+^CD68^+^ M2 macrophages. DES: drug-eluting scaffold; NES: non-eluting scaffold; Control: islets without scaffold

To investigate the underlying mechanism of the enhanced functional output of transplanted islets, we hypothesized that IL-4 accelerated the resolution of local inflammation by triggering the M2 polarization of macrophages. Therefore, we probed the presence and percentage of CD163^+^CD68^+^ M2 macrophages in the tissue. The flow cytometric analysis showed that, within 5 days after the islet transplantation, the islet tissues from mice in the drug-eluting scaffold group featured an increased presence of CD163^+^CD68^+^ M2 macrophages (Fig. 8). The percentages of CD163^+^CD68^+^ M2 macrophages were 2.75 ± 0.78% in the drug-eluting scaffold group and 1.22 ± 0.56% in the non-eluting scaffold group, respectively. This result confirmed that the locally released IL-4 molecules successfully steered the polarization of macrophages toward the M2 route.

## Discussion

Previous tissue engineering research has confirmed that the local milieu and stimulants play a governing role in the proliferation and functional output of in vivo tissue regeneration [13]. In light of previous research, we hereby attempted to offer the transplanted islets a favorable milieu to survive and proliferated in vivo by employing an electrospun scaffold. Both poliglecaprone (PGC) and collagen had been widely investigated for the use as tissue-engineered scaffolds in previous research [14, 15]. Both of them showed excellent biocompatibility, no cytotoxicity and tunable in vivo biodegradation profiles.

A chronic hyperglycemia is the primary cause for a variety of diseases in diabetic patients, such as delayed wound healing, nephrological hypertension, etc. Therefore, how to reduce and maintain the blood glucose level in the physiological range is the primary goal in the management of diabetes. In the last decade, electrospun scaffolds had been frequently re-tooled to carry a payload of biomolecules to stimulate the local tissues for enhanced therapeutic effect [12]. Consequently, in this study we attempted to enable the scaffold to carry a payload of IL-4 molecules. By employing the drug-eluting scaffold, we successfully enhanced the functional output of transplanted islets. In our study, the transplanted islets successfully survived and secreted sufficient insulin to reduce the blood glucose level. Furthermore, the OGTT result spoke to fact that the diabetic mice in the drug-eluting scaffold group possessed a strong capability to withstand sudden glucose spike. These results confirmed that our tissue engineering strategy achieved its overall therapeutic aim.

Macrophages are widely distributed immunological cells that play prominent roles ranging from the destruction of invading bacteria to the regulation of inflammation process [16]. Macrophages can be activated by two different routes, giving rise to two phenotypes, i.e. classically activated macrophages (M1) and alternatively activated macrophages (M2). The current academic consensus holds that M1 macrophages are considered to be pro-inflammatory phenotype, responsible for the initiation of inflammation against bacteria, virus, etc. On the other hand, M2 macrophages, an umbrella term for a handful of sub-phenotypes, are generally believed to be anti-inflammatory [17, 18]. The polarization of macrophages in the local tissues enables the immunological system to tightly regulate the inflammation process and the engagement with other immunological cells. Therefore, the manipulation of macrophages for therapeutic purposes has long been the primary goal of immunological research [19, 20]. In the inflammation site, both M1 and M2 macrophages are both present and the balance of them changes over the course of the immunological response. Previous research showed that contextual stimuli could artificially steer the activation of macrophages toward a certain route, a phenomenon called the polarization of macrophages [21]. M2 macrophages are generally considered to be CD163^+^CD68^+^ according to previous research [22].

Macrophages play a critical role in the local inflammation caused by the transplantation [23].

Consequently, we hypothesized that the enhanced functional output of transplanted islets might be attributed to the accelerated resolution of inflammation due to the M2 polarization of macrophages stimulated by the locally released IL-4 from the scaffold. The flow cytometric result showed that CD163^+^CD68^+^ M2 macrophages accounted for a higher percentage in the transplanted islet tissues from the drug-eluting scaffold group. This result suggested that IL-4 steered the polarization of macrophages toward the M2 anti-inflammatory route.

## Conclusion

Islet transplantation remains the most effective treatment for diabetic patients. Unfortunately, its functional output after the transplantation is far from being satisfactory. To boost the functional output, we developed a tissue engineering strategy to simultaneously provide the islets a favorable milieu and to suppress the local inflammation. The results showed that the use of an electrospun scaffold with a payload of IL-4 successfully enhanced the functional output of the transplanted islets. Through the manipulation of macrophages, therapeutic effect could be achieved without using pharmaceutical molecules that frequently cause significant side effects.

## Methods

### The scaffold fabrication and biophysical characterization

The scaffold was fabricated by electrospinning PGC (Advanced Inventory Management, Mokena, IL) and gelatin (Sigma-Aldrich, St Louis, MO). Both materials were fully dissolved in 1,1,1,3,3,3-hexafluoro-2-propanol (Sigma-Aldrich, St Louis, MO, USA) at a final weight/volume concentration of 14%. For drug-eluting scaffold, IL-4 (20 μg/mL) was added to the polymer solution. During the fabrication, 0.5 mL of the solution was electrospun at a feeding rate of 3 mL/h to a collector placed at 25 cm from the needle tip with the presence of a 30 kV voltage. Scaffolds were retrieved and desiccated in vacuum for 24 h prior to subsequent analyses. The morphology of the scaffold was investigated using scanning electron microscope (SEM). Diameters of electrospun fibers in respective scaffolds were measured in the SEM images.

### The in vitro studies of cytotoxicity

Islets were harvested from C57BL/6 mice and purified as previously reported [24, 25]. Desiccated scaffolds were cut into circular shape (D = 6.5 mm) and placed into 96-well plates. The scaffolds were sterilized with 70% ethanol at room temperature for 15 min followed by extensive rinse with sterile PBS buffer. Harvested islets were seeded onto non-eluting scaffolds, drug-eluting scaffolds and tissue culture plates (control) at a density of 100 islet equivalents per scaffold. The islets were incubated in the islet growth medium at 37 °C for two weeks. The viability of the islet population was measured using the tetrazolium compound [3-(4,5-dimethylthiazol-2-yl)-5-(3-carboxymethoxyphenyl)-2-(4-sulfophenyl)-2H-tetrazolium, inner salt] (MTS) (Promega Corporation, Madison, WI) reagent kit per manufacturer’s protocol. The apoptosis level of the islet population was measured using Caspase-Glo® 3/7 assay kit (Promega Corporation, Madison, WI) per manufacturer’s protocol.

### The in vitro pharmacokinetic study of IL-4 release

The drug-eluting scaffold (D = 1.6 cm) were incubated in sterile PBS at 37 °C and 5% CO_2_ for 7 days. The supernatant was collected day 1, 3, 5 and 7, and the concentration of IL-4 was measured using the Quantikine Mouse IL-4 Immunoassay kit (R&D System, Minneapolis, MN, U.S.). The non-eluting scaffold was used as control.

To measure the bioactivity of released IL-4, murine naïve CD4^+^ T cells were harvested from spleen tissues using an isolation kit per manufacturer’s protocol (Miltenyi Biotec, Auburn, CA, USA). Naïve CD4^+^ T cells were incubated with culture media containing IL-4 supernatant at 37 °C for 3 days. The expression of GATA-3 of CD4^+^ T cells was probed by flow cytometry using anti-GATA3 antibody per manufacturer’s protocol (Miltenyi Biotec, Auburn, CA, USA).

### The establishment of diabetic mice and islet transplantation

To induce diabetes, C57BL/6 mice (female, 6–10 week old) received a single intraperitoneal injection of streptozotocin (150 mg/kg, Sigma Chemical, St. Louis, MO). Those mice with non-fasted blood glucose level exceeding 400 mg/dL in at least two consecutive days was considered diabetic and used in subsequent studies. The animal study protocol was approved by Yinzhou People’s Hospital.

Islets were harvested and seeded on sterilized electrospun scaffolds (1 mm × 1 mm) at a density of 100 islet equivalents per scaffold. Then the islets on the scaffold were cultured in islet growth medium for 6 h at 37 °C and 5% CO_2_. During the transplantation surgery, diabetic mice were anesthetized using vaporized isoflurane. The islets together with the scaffold were surgically inserted into the renal capsule. In the control group, the islets were injected into the renal capsule without the support of the scaffold.

### The functional output analyses of transplanted islets

Blood glucose levels in mice were measured using OneTouch Ultra glucose tear (Lifescan, Johnson & Johnson, Milpitas, CA, U.S.). The blood samples were collected from the tail vein in non-fasted mice immediately prior to the surgery, and then on week 1, 2, 3 and 4. Serum insulin concentration was measured using a rat insulin enzyme-linked immunosorbent assay (ELISA) kit (Crystal Chem Inc. Chicago, IL, U.S.) whereas serum C-peptide 2 concentration using a rat C-peptide 2 immunoassay kit (Millipore, Bellirica, MA, U.S.) per manufacturer’s protocols.

OGTT was conducted in diabetic mice 2 weeks after the transplantation surgery. Prior to the test, mice were fasted for 16 h and the baseline glucose level was measured using blood samples collected from the tail vein. Then, glucose solution (100 mg/mL) was orally administered to each mouse at a dosage of 2 g/kg body weight. Following the glucose administration, blood glucose levels were measured at 30, 60 and 120 min using blood samples collected from the tail vein.

Renal tissues were harvested when mice were sacrificed at the end of week 8 after the surgery. Then renal tissues together with transplanted islets was embedded in Tissue-Tek OCT (Sakura Finetek, Torrance, CA), snap-frozen in liquid nitrogen, sliced and collected on charged glass slides. The samples were stained using anti-insulin antibody (Abcam, Cambridge, MA, USA) and hematoxylin.

### The polarization of macrophages in the transplantation site

At 7 days of the transplantation surgery, the diabetic mice were sacrificed and the transplanted islet tissues were retrieved. The islet tissue was broken down into single cell suspensions as previously reported [26]. Flow cytometry was used to probe the percentage of CD163^+^CD68^+^ M2 macrophages in the population using anti-CD163 antibody (Abcam, Cambridge, MA) and anti-CD68 antibody (Abcam, Cambridge, MA).

### Statistical analysis

ImageJ was used in all image processing. FlowJo was used to analyze the flow cytometric data and chart plotting. All data was analyzed using student t-test or ANOVA with a Tukey test with the significance level set at 95%.

## Abbreviations

ELISA: Enzyme-linked immunosorbent assay
MTS: Tetrazolium compound [3-(4,5-dimethylthiazol-2-yl)-5-(3-carboxymethoxyphenyl)-2-(4-sulfophenyl)-2H-tetrazolium, inner salt]
OGTT: Oral glucose tolerance test
PGC: Poliglecaprone
SEM: Scanning electron microscope
